# Is endovascular treatment still good for acute ischemic stroke in the elderly? A meta-analysis of observational studies in the last decade

**DOI:** 10.3389/fnins.2023.1308216

**Published:** 2024-01-05

**Authors:** Xin Jiang, Jian Wang, Yaowen Hu, Hui Lang, Jiajia Bao, Ning Chen, Li He

**Affiliations:** Department of Neurology, West China Hospital, Sichuan University, Chengdu, Sichuan, China

**Keywords:** acute ischemic stroke, clinical outcome, endovascular treatment, elderly, meta-analysis

## Abstract

**Background:**

The lack of randomized evidence makes it difficult to establish reliable treatment recommendations for endovascular treatment (EVT) in elderly patients. This meta-analysis aims to evaluate the therapeutic effects of endovascular treatment for acute ischemic stroke in the elderly compared with younger patients.

**Methods:**

Comprehensive literature retrieval was conducted to identify studies that directly compared the outcomes of EVT in elderly patients and those aged <80 years. The primary outcome was functional independence, defined as mRS 0–2 at 90 days after EVT. The secondary outcomes were the rate of successful recanalization, symptomatic intracranial hemorrhage (sICH) and mortality. Odds ratios (ORs) were estimated using a random effects model.

**Results:**

In total, twenty-six studies with 9,492 enrolled participants were identified. Our results showed that, compared with patients aged <80 years undergoing EVT, EVT was associated with a lower rate of functional independence at 90 days (OR = 0.38; 95% CI, 0.33–0.45; *p* < 0.00001) and a higher mortality rate (OR = 2.51; 95% CI, 1.98–3.18; p < 0.00001) in the elderly. Furthermore, even without a significantly observed increase in sICH (OR = 1.19; 95% CI, 0.96–1.47; *p* = 0.11), EVT appeared to be associated with a lower rate of successful recanalization (OR = 0.81; 95% CI, 0.68–0.96; *p* = 0.02).

**Conclusion:**

Evidence from observational studies revealed that EVT has less functional outcomes in elderly patients with acute ischemic stroke. Further studies are needed to better identify patients aged ≥80 years who could potentially benefit from EVT.

## Introduction

With the aging of the global population, the burden of cardiovascular and cerebrovascular diseases has increased significantly (Qi et al., 2023). Acute ischemic stroke, the most common and serious manifestation of cerebrovascular disease, is the leading cause of disability and death in adults in China (Liu et al., 2011; Wu et al., 2019; Tu and Wang, 2023). Endovascular treatment (EVT) has developed rapidly in the past decade and is now recommended as the standard reperfusion therapy for acute ischemic stroke (AIS) due to large vessel occlusion (LVO) (Powers et al., 2019; Herpich and Rincon, 2020; Wassélius et al., 2022). Even with advances in technology and improved recanalization rates, only half of patients who received EVT could regain functional independence (Goyal et al., 2016; Pajor and Adeoye, 2023). However, patients who received EVT in real world practice substantially differ from those fulfilling trial inclusion criteria. In the past few years, much effort has been made to promote trials examining EVT efficacy and safety in various conditions, including extended time window, large ischemic core, distal occlusions and more (Rikhtegar et al., 2021; Kobeissi et al., 2023; Sarraj et al., 2023a,b). Patients aged ≥80 years, accounting for over 30% of stroke admissions, are excluded from several landmark clinical trials due to very stringent inclusion/exclusion criteria necessitating good functional baseline, and only a small number of patients enrolled in the remainder (Chen et al., 2015). This is likely related to the fact that elderly patients are more likely to experience poor functional outcome and complications, such as intracranial hemorrhage and infection (Fonarow et al., 2010).

Many observational studies have investigated the safety and efficacy of EVT in elderly patients and the conclusions are inconsistent (Alawieh et al., 2018; Adcock et al., 2022). According to the results of a previous meta-analysis, the proportion of functional independence at 90 days after EVT in elderly patients was only 27% (Hilditch et al., 2018). In addition, the results of two meta-analyses in 2019 showed that elderly patients who underwent EVT had a worse functional outcome and higher mortality, and there was a trend toward an increased incidence of symptomatic intracranial hemorrhage (sICH) and a decreased successful recanalization (Sharobeam et al., 2019; Zhao et al., 2019). We noticed that, in recent years, many new studies have done effort in investigating the efficacy and safety of EVT in elderly patients and the results are discrepant (Choi et al., 2021; Jiao et al., 2022; Narloch et al., 2023; Scopelliti et al., 2023). Some studies indicated that elderly patients undergoing EVT had comparable functional outcomes and rates of successful recanalization to younger patients, without an increased risk of sICH, while others studies failed to confirm this conclusion (Kawabata et al., 2019; Groot et al., 2020; Choi et al., 2021; Sudre et al., 2021; Han et al., 2023; Narloch et al., 2023). Considering that it has been more than four years since the latest meta-analysis by Zhao et al., we sought to conduct this meta-analysis of available observational studies published in the last decade to evaluate the therapeutic effects of endovascular treatment for acute ischemic stroke in the elderly compared with younger patients.

## Methods

### Literature search

Literatures were systematically searched by 2 reviewers (XJ and JW) in PubMed, EMBASE, and Cochrane Library (Cochrane Database of Systematic Reviews, Cochrane Central Register of Controlled Trials, Cochrane Methodology) from September 2013 to September 2023. For the search strategy, the following keywords and free text searches were used in combination with the Boolean operators “or” and “and”: acute ischemic stroke, large vessel occlusion, mechanical thrombectomy, thrombectomy, endovascular therapy, endovascular treatment, elderly, octogenarian, nonagenarian, 80 or older. This meta-analysis was conducted according to the recommendations of the preferred reporting items for systematic reviews and meta-analyses (PRISMA) guidelines (Moher et al., 2009).

### Selection criteria

We included studies comparing outcomes of endovascular treatment for acute ischemic stroke in the elderly and in patients younger than 80 years of age. Full texts of eligible studies were reviewed according to the criteria of inclusion and exclusion. Disagreements were resolved by consensus. The inclusion criteria were as follows: (1) studies reporting patients with acute ischemic stroke who received EVT; (2) studies reporting results of clinical follow-up, especially functional independence at 90 days; and (3) studies with direct comparison of clinical outcome between elderly and young patients who were under 80 years old. Those with <10 participants in either group or those lacking outcome variables, especially the modified Rankin Scale (mRS) at 90 days, were excluded.

### Data extraction and quality assessment

Two authors (XJ and YH) separately reviewed all eligible articles and extracted data using a structured data extraction form. The following data were extracted: (1) study characteristics: first author, year of publication, study design, sample size, and quality of study; (3) data relating to treatment: admission NIHSS score, functional independence at 90 days, mortality, sICH, and successful recanalization rate. The risk of bias was assessed by 2 reviewers independently. The Newcastle-Ottawa Scale (NOS) was used to assess the quality of each eligible study (Stang, 2010).

### Outcome measures

The proportion of patients with mRS scores 0 to 2 at 90 days after endovascular treatment was considered the primary outcome (Banks and Marotta, 2007). The secondary outcomes included successful recanalization rate, sICH, and mortality at 90 days (Von Kummer et al., 2015).

### Statistical analysis

The statistical analysis was performed by using Review Manager 5.4 software. Odds ratios (ORs) with 95% CIs were calculated and pooled for each outcome of interest. As clinical diversity and methodological differences among the studies were assumed, a random-effects model was used to pool outcomes for all meta-analyses (DerSimonian and Laird, 1986). The statistical heterogeneity between studies was assessed using the Q test and the calculation of I^2^. We considered *p* < 0.10 or I^2^ ≥ 50% as an indication of substantial heterogeneity. We used subgroup analysis to analyze the source of heterogeneity when I^2^ ≥ 40% (Cumpston et al., 2022). Visual funnel plots were used to evaluate the publication bias in this meta-analysis.

### Results

**Figure 1:**
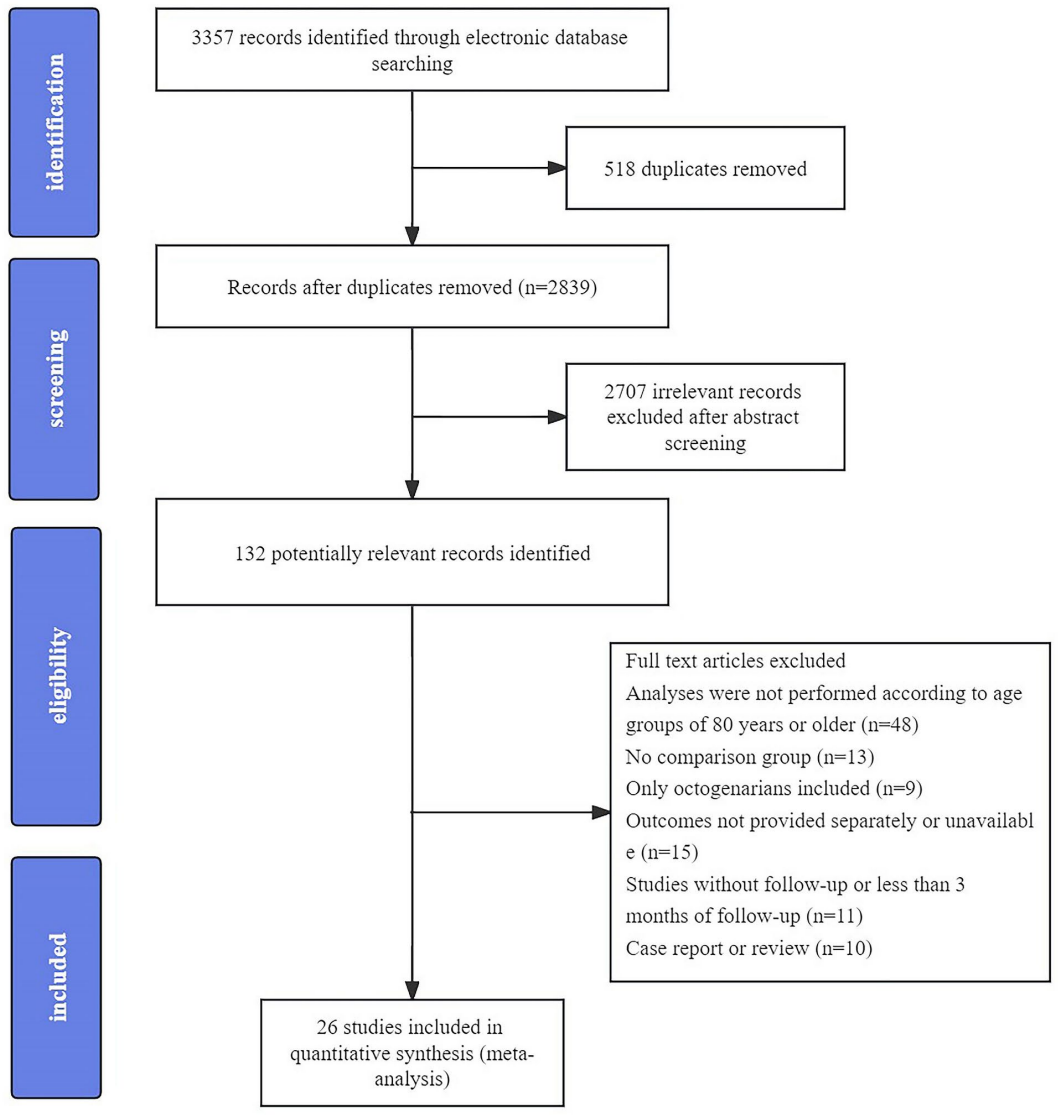
PRISMA flowchart of study selection.

The initial literature search yielded 3,357 articles and 26 studies of 9,492 enrolled participants (2,303 ≥ 80 years of age and 7,189 below 80 years) were finally included in this meta-analysis after screening the abstract and full text (Castonguay et al., 2014; Parrilla et al., 2015; Broussalis et al., 2016; Cohen et al., 2016; Kleine et al., 2016; Calle et al., 2017; Figueiredo et al., 2017; Imahori et al., 2017; Sallustio et al., 2017; Son et al., 2017; Tajima et al., 2017; Alawieh et al., 2018; Karhi et al., 2018; Koizumi et al., 2018; Alawieh et al., 2019; Kawabata et al., 2019; Rezai et al., 2019; Sharobeam et al., 2019; Groot et al., 2020; Choi et al., 2021; Sudre et al., 2021; Jiao et al., 2022; Han et al., 2023; Narloch et al., 2023; Rhiner et al., 2023; Scopelliti et al., 2023). A flow diagram of the detailed search process is presented in Figure 1. Among the included studies, eight were multicenter studies, and the others were single-center studies. The detailed baseline characteristics and outcomes of each study are presented in Supplemental Table S1.

### mRS score 0 to 2 at 90 days

**Figure 2:**
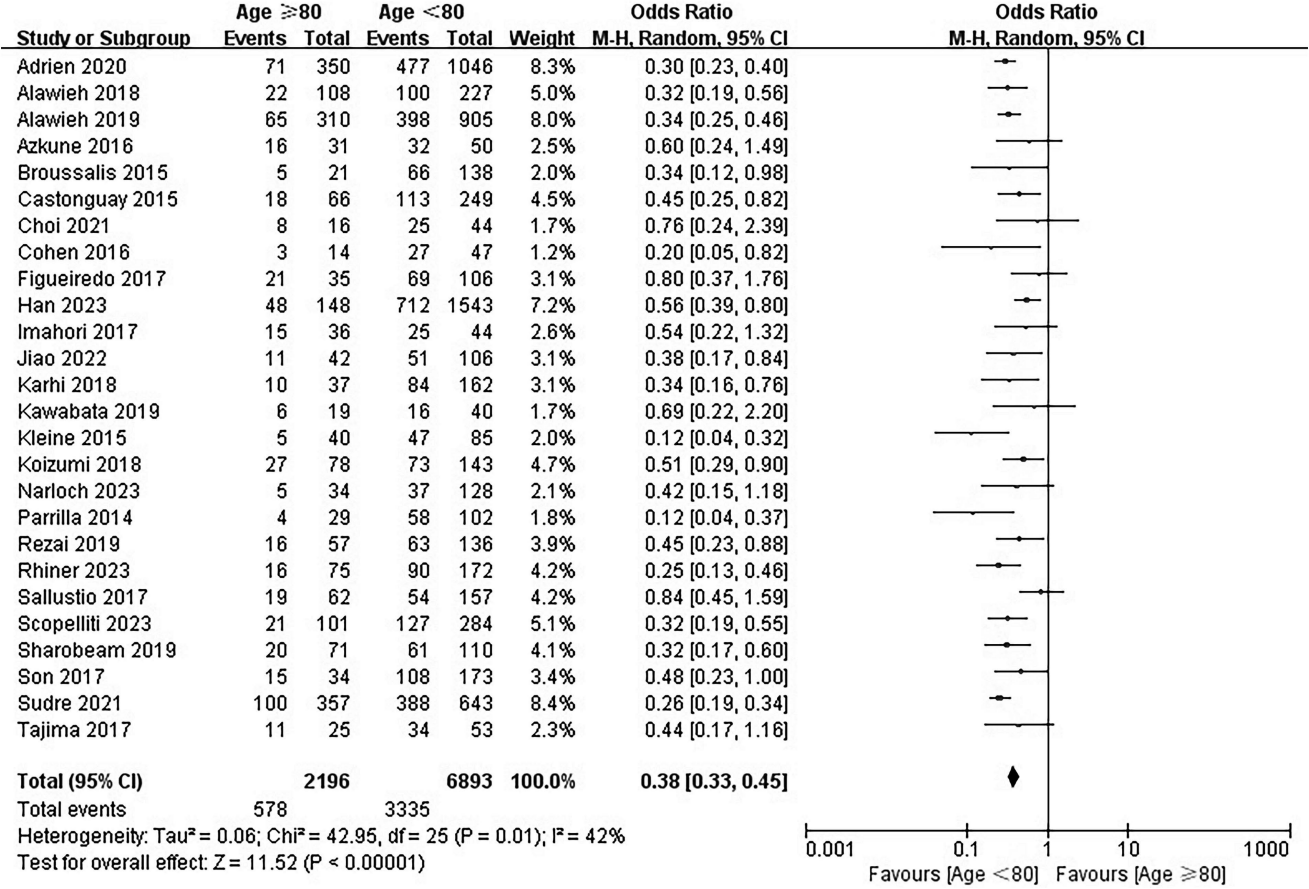
Forest plot of modified Rankin Scale score of 0–2 at 90 days.

For the primary outcome, 403 of included patients were lost to follow-up at 90 days, and 9,089 patients were included in the analysis. The rates of mRS scores of 0–2 were 26.3% (578/2196) and 48.4% (3,335/6893) in the elderly and younger arms, respectively. In the main analysis, EVT was associated with lower odds of functional independence in elderly patients (OR = 0.38; 95% CI, 0.33–0.45; *p* < 0.00001; Figure 2) than in young patients. Substantial heterogeneity was detected (I^2^ = 42%) and multiple subgroup analyses were performed.

In the additional analysis, EVT in elderly patients was associated with worse functional outcomes in both multicenter studies (OR = 0.39; 95% CI, 0.32–0.47) and single-center studies (OR = 0.38; 95% CI, 0.29–0.49) (Supplemental Figure S1). Moreover, no observed difference was identified in different study period subgroups (OR = 0.42, 95% CI 0.32–0.55 for studies published between 2014–2018 and OR = 0.35, 95% CI 0.29–0.42 for studies published between 2019–2023) (Supplemental Figure S2).

### Successful recanalization rate

**Figure 3:**
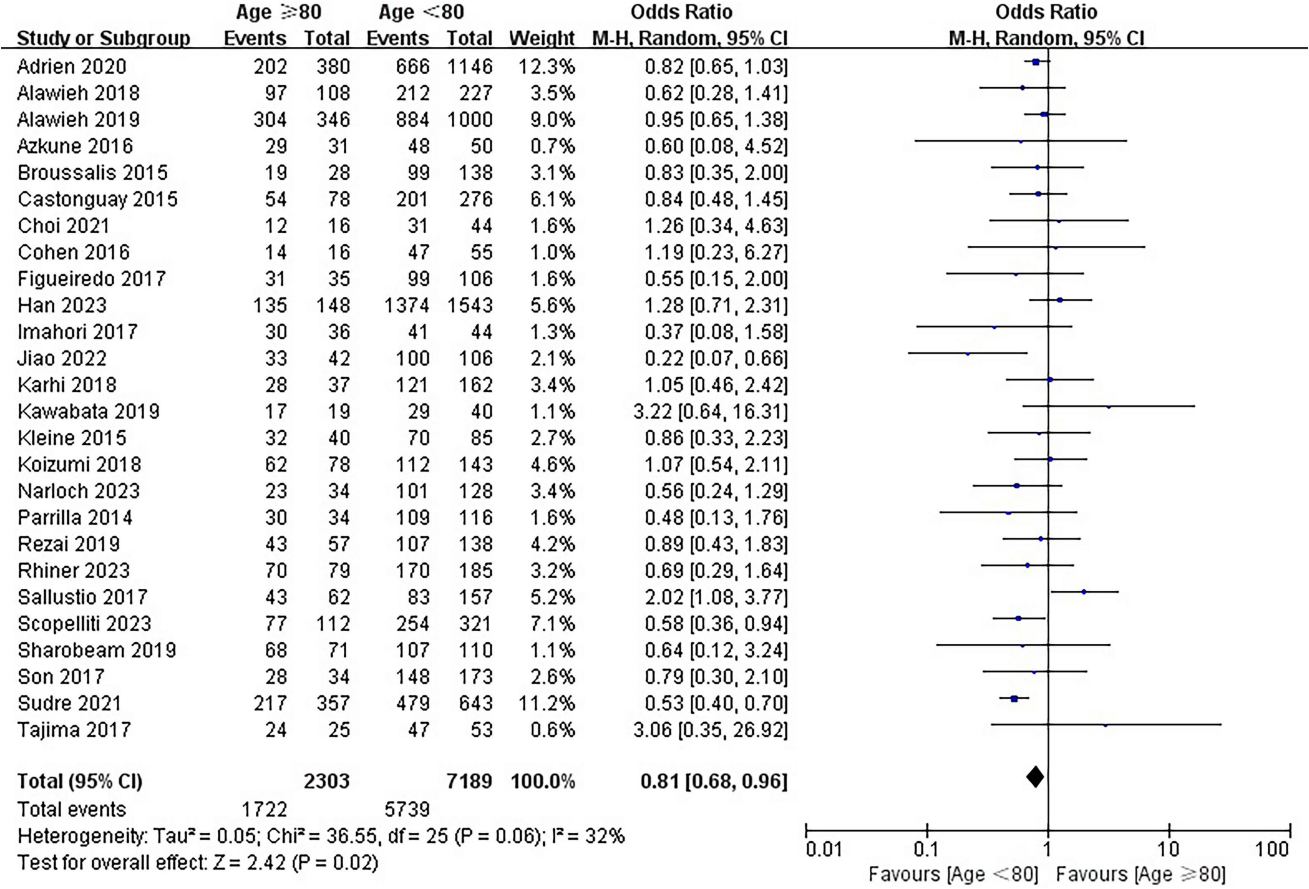
Forest plot of successful recanalization rate.

The rates of successful recanalization were 74.8% (1722/2303) and 79.8% (5,739/7189) in the elderly and younger arms, respectively. The results of the analysis showed that EVT was associated with a lower recanalization rate in the elderly (OR = 0.81; 95% CI, 0.68–0.96; *p* = 0.02; Figure 3). No substantial heterogeneity was detected across the 26 included studies (I^2^ = 32, *p* = 0.06).

### Symptomatic intracranial hemorrhage

**Figure 4:**
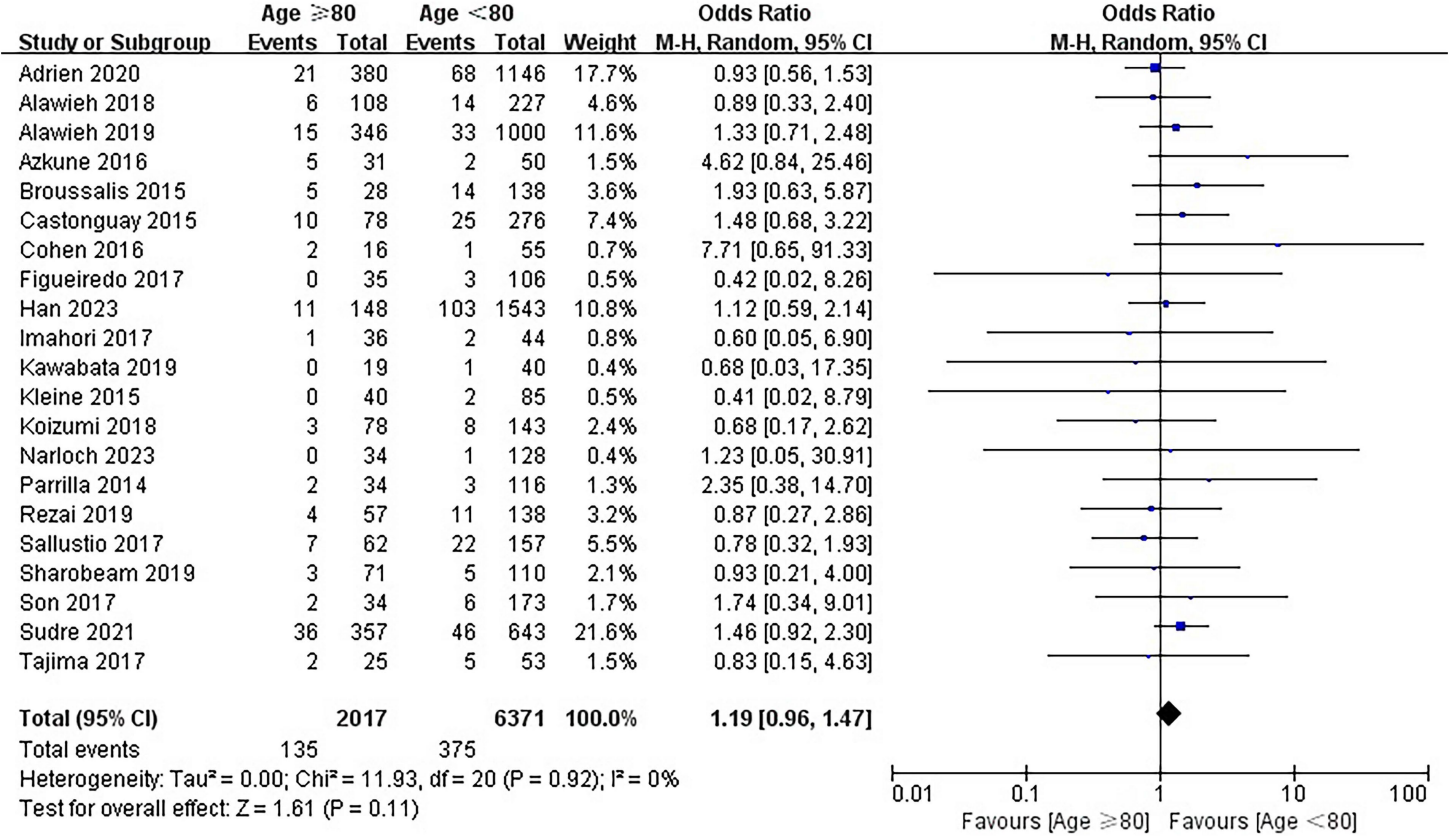
Forest plot of symptomatic intracranial hemorrhage (sICH).

Twenty-one studies involving 8,388 patients reported the information regarding sICH. The rates of sICH were 6.7% (135/2017) and 5.9% (375/6371) in the elderly and younger arms, respectively. No significant difference was identified between the elderly group and young group (OR = 1.19; 95% CI, 0.96–1.47; *p* = 0.11; Figure 4). No heterogeneity among the 21 studies was identified (*p* = 0.92, I^2^ = 0%).

### Mortality

**Figure 5:**
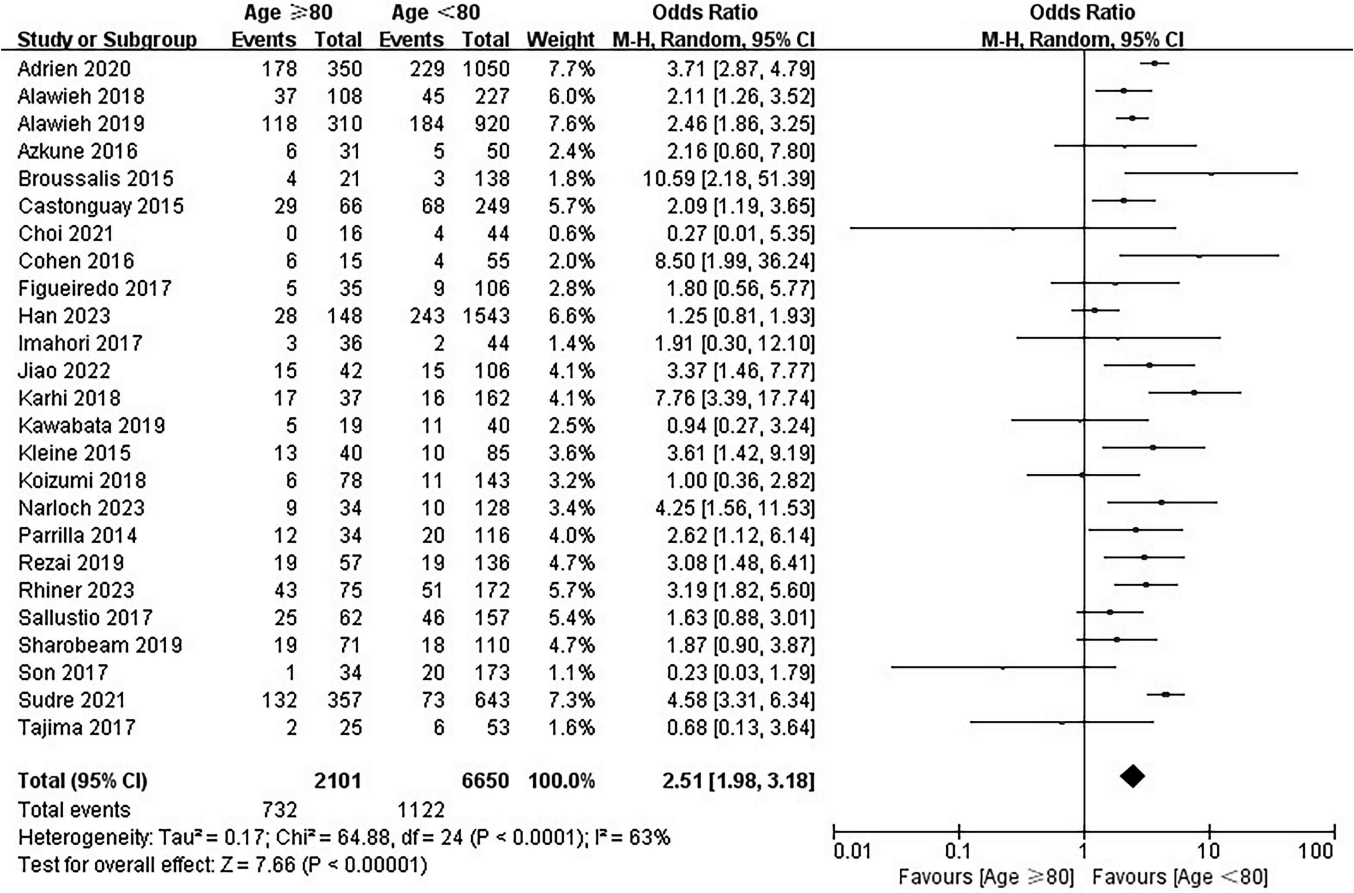
Forest plot of mortality at 90 days.

Twenty-five studies involving 8,751 patients reported information regarding mortality at 90 days. The overall mortality was 34.8% (732/2101) and 16.9% (1,122/6650) in the elderly and younger arms, respectively. In the main analysis, EVT was associated with a significantly higher mortality in the elderly (OR = 2.51; 95% CI, 1.98–3.18; *p* < 0.00001; Figure 5). Substantial heterogeneity was detected (I^2^ = 63%) and multiple subgroup analyses were performed. In the additional analysis, similar results were observed in both the study design subgroup (OR = 2.92, 95% CI 2.14–3.98 for single-center studies and OR = 1.99, 95% CI 1.38–2.87 for multicenter studies) and the study period subgroup (OR = 2.36, 95% CI 1.61–3.46 for studies published between 2014–2018 and OR = 2.65, 95% CI 1.95–3.60 for studies published between 2019–2023) (Supplemental Figure S3, S4).

### Quality and bias assessment

The quality of 5 of 26 studies was calculated as 6 by the Newcastle-Ottawa Scale, due to unclear selection methods, insufficient follow-up, and low comparability of the studies (Supplemental Table S2). No significant publication bias was observed in funnel plots among the included studies (Supplemental Figures S5–S8).

## Discussion

In this study, we made a direct comparison showing that, compared with patients younger than 80 years undergoing EVT, EVT was associated with lower rate of functional independence at 90 days and higher mortality in the elderly. Furthermore, even without a significantly observed increase in sICH, EVT appeared to be associated with a lower rate of successful recanalization. Our results are consistent with previous meta-analyses and, to the best of our knowledge, this study represents the largest comprehensive meta-analysis of the data including 26 studies of 9,492 enrolled participants evaluating the therapeutic effect of endovascular treatment in the elderly (Sharobeam et al., 2019; Zhao et al., 2019).

It is well established that advanced age is an independent predictor of poor prognosis in LVO patients who underwent EVT (Russo et al., 2011; Saposnik et al., 2019). Although endovascular treatment has shown remarkable effects in patients with large vessel occlusion stroke, the effectiveness is limited to a selected population (Widimsky et al., 2023). The proportion of functional independence for endovascular treatment in elderly patients reported in randomized controlled trials was 29.8%, vs. 13.9% in those who did not receive EVT (Goyal et al., 2016). In our study, the proportion of functional independence in elderly patients who received EVT is 26.3%, which is consistent with previous study (Hilditch et al., 2018). The reasons are multiple and there are several possible explanations for the poor prognosis in the elderly. First and foremost, age is closely linked with frailty, which has been proven to be independently associated with poor functional outcome (Pinho et al., 2021; Tan et al., 2022). Second, people with advanced age may have a decreased neurological reserve and a higher risk of decompensation of previously stable or nondisabling conditions than young patients, which may delay neurologic recovery (Chandra et al., 2012; Imam et al., 2021). In addition, elderly patients are reported to have higher rates of in-hospital complications, a higher incidence of health care–associated infections, and a higher risk of cognitive decline (Denti et al., 2010; Robertson et al., 2013; Cosentino et al., 2021). To explore whether the advances of EVT techniques and nursing management had an impact on the results, we further conducted subgroup analyses based on study period. The results indicated no observed difference between studies published in 2014–2018 and 2019–2023. Moreover, our results showed that elderly patients who received EVT were associated with a lower rate of successful recanalization, thus affecting the prognosis of neurological function (Groot et al., 2020). The lower rate of successful recanalization in elderly patients may be due to severe vascular stiffness and tortuosity, which could increase the difficulty of the operation to some extent (Shirakawa et al., 2017; Koge et al., 2022; Bala et al., 2023). In addition, elderly patients may receive lower number of thrombectomy passes, which might reduce the likelihood of recanalization (Alawieh et al., 2019). Moreover, a recent study revealed that aspiration was associated with better reperfusion in elderly patients compared with stent-retriever, the use of EVT technique in different studies may have an impact on this difference (D’Anna et al., 2023). However, since the number of thrombectomy passes and devices of EVT could not be extracted from all included studies, it is impossible to perform further analysis to confirm our presumption.

Our study provides the latest evidence from observational studies that patients aged ≥80 years with LVO stroke undergoing endovascular treatment had a worse neurological outcome, higher risk of mortality, and lower rate of successful recanalization than young patients. Our results have implications for clinical practice and future research. First, EVT should be offered to elderly patients with acute ischemic stroke due to a significantly observed improvement in functional outcome compared with natural history. However, patient selection should be prudent and the evaluation before EVT should be adequate. Ten of the included studies enrolled patients with premorbidly functional dependence (mRS ˃2), and the proportion was higher in elder patients in most of these studies. Further studies are needed to better estimate the therapeutic effect of EVT and best EVT technique (i.e., aspiration vs. stent retriever), and to better identify elderly patients who will not benefit from recanalization (i.e., pre-mRS, baseline NIHSS score, ASPECTS, type of anesthesia, leukoaraiosis, and brain atrophy) to avoid futile recanalization and potentially harmful interventions.

There are some limitations that need to be acknowledged. First, all the studies included in this meta-analysis were observational in nature, and the associations of EVT with clinical outcomes in elderly patients were determined in a nonrandomized manner. Selection bias in the treatment of patients in clinical practice is inevitable. Second, the heterogeneity was substantial in some outcomes, although the risk of bias was reasonably excluded. Furthermore, more detailed data could not be completely extracted from the included studies, including the type of anesthesia, number of procedures, time from puncture to recanalization, complications, etc., and part of the analysis could not be performed.

## Conclusion

This meta-analysis provides supporting evidence based on real-world data that EVT is associated with a lower rate of functional recovery and successful recanalization, and a higher risk of mortality in elderly patients. Further studies are needed to better identify patients aged ≥80 years who could potentially benefit from EVT.

## Data availability statement

The original contributions presented in the study are included in the article/Supplementary material, further inquiries can be directed to the corresponding authors.

## Author contributions

XJ: Conceptualization, Data curation, Formal Analysis, Investigation, Methodology, Software, Writing – original draft. JW: Conceptualization, Formal Analysis, Investigation, Methodology, Writing – original draft. YH: Data curation, Formal Analysis, Methodology, Writing – original draft. HL: Data curation, Methodology, Writing – original draft. JB: Data curation, Methodology, Writing – original draft. NC: Conceptualization, Data curation, Formal Analysis, Investigation, Methodology, Software, Supervision, Visualization, Writing – original draft. LH: Conceptualization, Data curation, Formal Analysis, Investigation, Methodology, Project administration, Resources, Software, Supervision, Visualization, Writing – original draft.
