# Supplementary material for: Is endovascular treatment still good for acute ischemic stroke in the elderly? A meta-analysis of observational studies in the last decade
Source: Front Neurosci. 2024 Jan 5;17:1308216. doi: 10.3389/fnins.2023.1308216 (PMC10796798; doi:10.3389/fnins.2023.1308216)

Supplemental Table S1: **Characteristics of studies included in this meta-analysis.**

Study	Design	Age ≥80							Age <80						
		No. of patients	Admission NIHSS score	Use of IV rt-PA	Successful recanalization	mRS ≤2 at 3 month	sICH	Mortality	No. of patients	Admission NIHSS score	Use of IV rt-PA	Successful recanalization	mRS ≤2 at 3 month	sICH	Mortality
Adrien 2020 *	Retrospective multicenter	380	17	290 (54.7)	202 (54.2)	71/350 (20.3)	21 (5.5)	178/350 (50.9)	1146	15	880 (77.0)	666 (58.9)	477/1046 (45.6)	68 (5.9)	229/1050 (21.8)
Alawieh 2018	Retrospective multicenter	108	17.3	49 (45.3)	97 (89.8)	22 (20.5)	6 (5.6)	37 (34.3)	227	15.8	81 (36)	212 (94.2)	100 (44.4)	14 (6.2)	45 (20)
Alawieh 2019 *	Retrospective multicenter	346	17	168 (49)	304 (88)	65/310 (21)	15 (7)	118/310 (38)	1000	16	445 (45)	884 (88)	398/905 (44)	33 (4)	184/920 (20)
Azkune 2016	Retrospective single-center	31	16.6	8 (25.8)	29 (93.6)	16 (51.6)	5 (16.1)	6 (19.4)	50	13.6	19 (38.0)	48 (96.0)	32 (64.0)	2 (4.0)	5 (10.0)
Broussalis 2015 *	Retrospective single-center	28	18	14 (50)	19 (68)	5/21 (24)	5 (18)	4/21 (19)	138	18	86 (62)	99 (72)	66 (48)	14 (10)	3 (2)
Castonguay 2015 *	Retrospective multicenter	78	18.9	37 (47.4)	54 (69.2)	18/66 (27.3)	10 (12.8)	29/66 (43.9)	276	17.9	117 (42.6)	201 (73.1)	113/249 (45.4)	25 (9.1)	68/249 (27.3)
Choi 2021	Retrospective single-center	16	12.56	5 (31.3)	12 (75.0)	8 (50.0)	NA	0 (0)	44	11.0	21 (47.7)	31 (70.5)	25 (56.8)	NA	4 (9.1)
Cohen 2016 *	Retrospective single-center	16	18.4	7 (44)	14 (88)	3/14 (21)	2 (13)	6/15 (40)	55	18.2	21 (38)	47 (87)	27/47 (57)	1 (2)	4 (7)

Figueiredo 2017	Retrospective single-center	35	17	24 (68.6)	31 (88.6)	21 (60)	0	5 (14.3)	106	17	83 (79)	99 (93.4)	69 (65.1)	3 (2.8)	9 (8.5)
Han 2023	Retrospective multicenter	148	18	NA	135 (91.2)	48 (32.4)	11 (7.6)	28 (18.9)	1543	16	NA	1374 (89.05)	712 (46.1)	103 (6.97)	243 (15.6)
Imahori 2017	Retrospective single-center	36	15	6 (17)	30 (83)	15 (42)	1 (3)	3 (8)	44	15	12 (27)	41 (93)	25 (57)	2 (5)	2 (5)
Jiao 2022	Retrospective single-center	42	18	9 (21.4)	33 (78.6)	11 (26.2)	NA	15 (35.7)	106	15	44 (41.5)	100 (94.3)	51 (48.1)	NA	15 (14.2)
Karhi 2018	Retrospective single-center	37	14.1	21 (56.8)	28 (75.7)	10 (27.0)	NA	17 (45.9)	162	12.7	109 (67.3)	121 (74.7)	84 (51.9)	NA	16 (9.9)
Kawabata 2019	Retrospective multicenter	19	23.42	13 (68.4)	17 (89.5)	6 (31.6)	0	5 (21.1)	40	20.4	21 (52.5)	29 (67.5)	16 (40)	1 (2.5)	11 (27.5)
Kleine 2015	Retrospective single-center	40	15	29 (73)	32 (80)	5 (12)	0	13 (33)	85	15	54 (64)	70 (82.4)	47 (55)	2 (2.4)	10 (12)
Koizumi 2018	Retrospective multicenter	78	19	41 (53)	62 (79)	27 (35)	3 (4)	6 (8)	143	17	73 (51)	112 (78)	73 (51)	8 (6)	11 (8)
Narloch 2023	Retrospective single-center	34	15.5	21 (62)	23 (67.6)	5 (14.7)	0	9 (26.4)	128	16	87 (68)	101 (78.9)	37 (28.9)	1 (0.8)	10 (7.8)
Parrilla 2014 *	Retrospective single-center	34	18.1	11 (32.4)	30 (88.2)	4/29 (13.8)	2 (5.9)	12 (35.3)	116	16.8	56 (48.3)	109 (93.9)	58/102 (56.9)	3 (2.6)	20 (17.2)
Rezai 2019 *	Prospective single-center	57	19	30 (53)	43 (75)	16 (28)	4 (7)	19 (33)	138	17	100 (72)	107 (78)	63/136 (46)	11 (8)	19/136 (14)

Rhiner 2023 *	Retrospective single-center	79	13.5	22 (27.8)	70 (88.6)	16/75 (21.3)	NA	43/75 (57.3)	185	12	54 (29.2)	170 (91.9)	90/172 (52.3)	NA	51/172 (29.7)
Sallustio 2017	Retrospective single-center	62	18	42 (67.7)	43 (69)	19 (30.6)	7 (11)	25 (40.3)	157	18	83 (52.8)	99 (63)	54 (34.3)	22 (14)	46 (29.2)
Scopelliti 2023 *	Retrospective multicenter	112	20	65 (58.6)	77 (68.8)	21/101 (20.8)	NA	NA	321	17	218 (68.1)	254 (79.1)	127/284 (44.7)	NA	NA
Sharobeam 2019	Retrospective single-center	71	18	24 (34)	68 (96)	20 (28)	3 (4)	19 (27)	110	17	49 (45)	107 (97)	61 (55)	5 (5)	18 (16)
Son 2017	Retrospective single-center	34	18	11 (32.4)	28 (82.4)	15 (44.1)	2 (5.9)	1 (2.9)	173	16	80 (46.2)	148 (85.5)	108 (62.4)	6 (3.5)	20 (11.6)
Sudre 2021	Retrospective single-center	357	15	252 (70.6)	217 (75.1)	100 (28.0)	36 (10.1)	132 (37.1)	643	12	470 (73.1)	438 (86.0)	388 (60.3)	46 (7.1)	73 (11.4)
Tajima 2017	Retrospective single-center	25	22.4	6 (25)	24 (96)	11 (44)	2 (8)	2 (8)	53	20.2	22 (41.5)	47 (88.7)	34 (64.2)	5 (9.4)	6 (11.3)

Footnote:

NIHSS: National Institutes of Health Stroke Scale, mRS: modified Rankin Scale, sICH: symptomatic intracranial hemorrhage

* Study with some loss to follow-up at 3 months.

Supplemental Table S2: **Quality assessment of studies included for meta-analysis.**

Study	Selection				Comparability	Outcome			Score
	Representativeness of the exposed cohort	Selection of the nonexposed cohort	Ascertainment of exposure	Demonstration that outcome of interest was not present at start of study	Comparability of controls on the basis of the design or analysis	Assessment of outcome	Was follow up long enough for outcomes to occur	Adequacy of follow up of cohorts	Total scores
Adrien 2020	1	1	1	1	1	1	1	0	7
Alawieh 2018	1	1	1	1	1	1	1	1	8
Alawieh 2019	1	1	1	1	1	1	1	1	8
Azkune 2017	1	0	1	1	1	1	1	1	7
Broussalis 2015	1	0	1	1	1	1	1	0	6
Castonguay 2014	1	0	1	1	1	1	1	1	7
Choi 2021	1	0	1	1	1	1	1	1	7
Cohen 2016	1	1	1	1	1	0	1	0	6
Figueiredo 2017	1	0	1	1	1	1	1	1	7
Han 2023	1	1	1	1	1	1	1	1	8

Imahori 2017	0	0	1	1	1	1	1	1	6
Jiao 2022	1	1	1	0	1	1	1	1	7
Karhi 2018	1	1	0	1	1	1	1	1	7
Kawabata 2019	1	0	1	0	1	1	1	1	6
Kleine 2016	1	1	0	1	1	1	1	1	7
Koizumi 2018	1	1	1	0	1	1	1	1	7
Narloch 2023	1	0	1	0	1	1	1	1	6
Parrilla 2015	1	1	1	1	1	1	1	0	7
Rezai 2019	1	1	1	1	1	1	1	1	8
Rhiner 2023	1	0	1	1	1	1	1	1	7
Sallustio 2017	1	1	1	1	1	1	1	1	8
Scopelliti 2023	1	1	1	1	1	1	1	0	7
Sharobeam 2019	1	0	1	1	1	1	1	1	7
Son 2017	1	1	1	1	1	1	1	1	8
Sudre 2021	1	1	1	1	1	1	1	1	8
Tajima 2017	1	1	1	1	1	1	1	1	8

Supplemental Figure S1: Forest plot of subgroup analyses of functional outcome according to study design

Study or Subgroup	Age ≥80 Events	Age ≥80 Total	Age <80 Events	Age <80 Total	Weight	Odds Ratio M-H, Random, 95% CI
**1.6.1 single-center**						
Azkune 2016	16	31	32	50	2.5%	0.60 [0.24, 1.49]
Broussalis 2015	5	21	66	138	2.0%	0.34 [0.12, 0.98]
Choi 2021	8	16	25	44	1.7%	0.76 [0.24, 2.39]
Cohen 2016	3	14	27	47	1.2%	0.20 [0.05, 0.82]
Figueiredo 2017	21	35	69	106	3.1%	0.80 [0.37, 1.76]
Imahori 2017	15	36	25	44	2.6%	0.54 [0.22, 1.32]
Jiao 2022	11	42	51	106	3.1%	0.38 [0.17, 0.84]
Karhi 2018	10	37	84	162	3.1%	0.34 [0.16, 0.76]
Kleine 2015	5	40	47	85	2.0%	0.12 [0.04, 0.32]
Narloch 2023	5	34	37	128	2.1%	0.42 [0.15, 1.18]
Parrilla 2014	4	29	58	102	1.8%	0.12 [0.04, 0.37]
Rezai 2019	16	57	63	136	3.9%	0.45 [0.23, 0.88]
Rhiner 2023	16	75	90	172	4.2%	0.25 [0.13, 0.46]
Sallustio 2017	19	62	54	157	4.2%	0.84 [0.45, 1.59]
Sharobeam 2019	20	71	61	110	4.1%	0.32 [0.17, 0.60]
Son 2017	15	34	108	173	3.4%	0.48 [0.23, 1.00]
Sudre 2021	100	357	388	643	8.4%	0.26 [0.19, 0.34]
Tajima 2017	11	25	34	53	2.3%	0.44 [0.17, 1.16]
**Subtotal (95% CI)**		**1016**		**2456**	**55.5%**	**0.38 [0.29, 0.49]**
Total events	300		1319			
Heterogeneity: Tau² = 0.12; Chi² = 31.95, df = 17 (P = 0.02); I² = 47%						
Test for overall effect: Z = 7.60 (P < 0.00001)						
**1.6.2 multicenter**						
Adrien 2020	71	350	477	1046	8.3%	0.30 [0.23, 0.40]
Alawieh 2018	22	108	100	227	5.0%	0.32 [0.19, 0.56]
Alawieh 2019	65	310	398	905	8.0%	0.34 [0.25, 0.46]
Castonguay 2015	18	66	113	249	4.5%	0.45 [0.25, 0.82]
Han 2023	48	148	712	1543	7.2%	0.56 [0.39, 0.80]
Kawabata 2019	6	19	16	40	1.7%	0.69 [0.22, 2.20]
Koizumi 2018	27	78	73	143	4.7%	0.51 [0.29, 0.90]
Scopelliti 2023	21	101	127	284	5.1%	0.32 [0.19, 0.55]
**Subtotal (95% CI)**		**1180**		**4437**	**44.5%**	**0.39 [0.32, 0.47]**
Total events	278		2016			
Heterogeneity: Tau² = 0.02; Chi² = 10.43, df = 7 (P = 0.17); I² = 33%						
Test for overall effect: Z = 9.47 (P < 0.00001)						
**Total (95% CI)**		**2196**		**6893**	**100.0%**	**0.38 [0.33, 0.45]**
Total events	578		3335			
Heterogeneity: Tau² = 0.06; Chi² = 42.95, df = 25 (P = 0.01); I² = 42%						
Test for overall effect: Z = 11.52 (P < 0.00001)						
Test for subgroup differences: Chi² = 0.02, df = 1 (P = 0.88), I² = 0%						

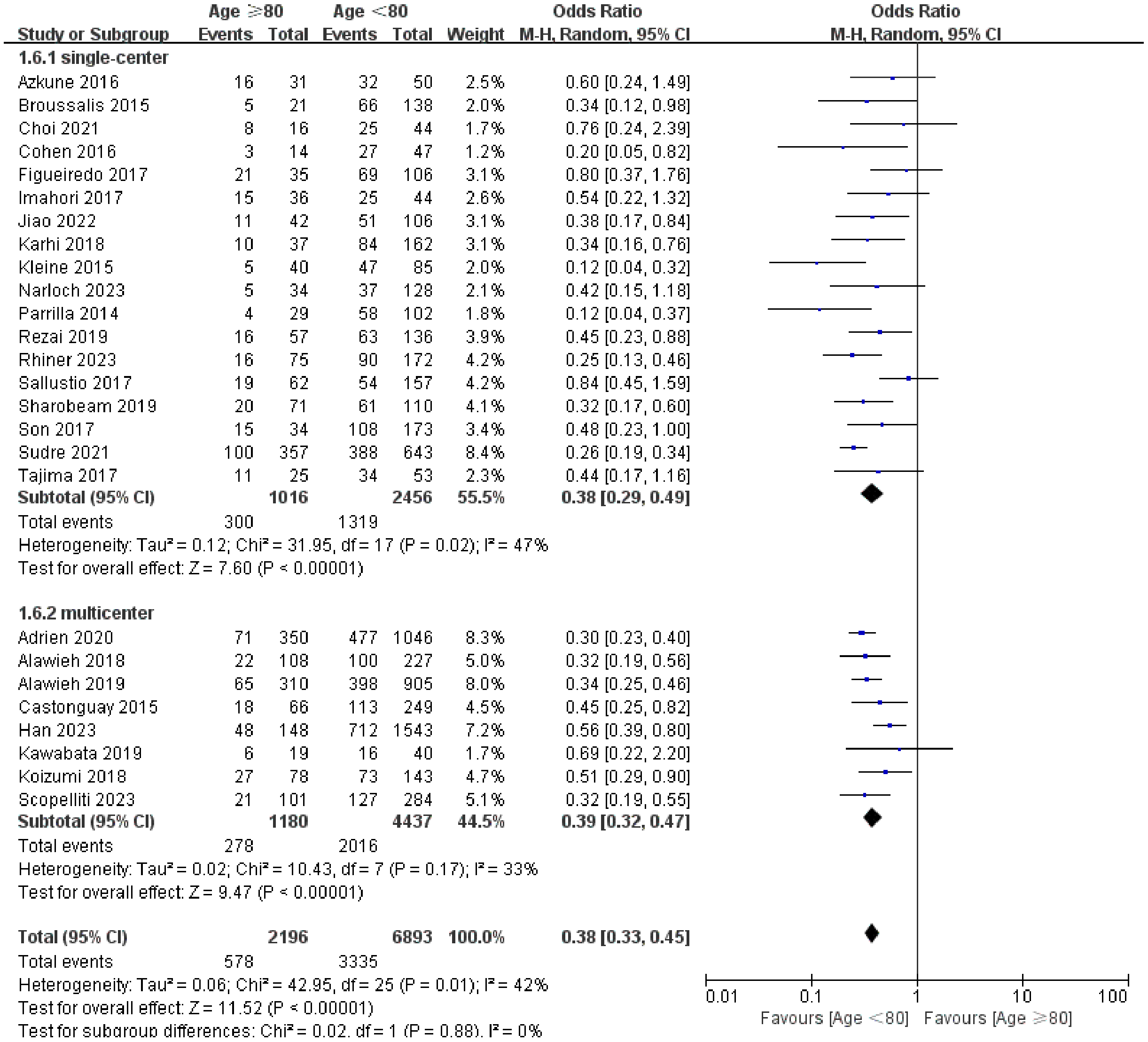

Supplemental Figure S2: Forest plot of subgroup analyses of functional outcome according to study period

Study or Subgroup	Age ≥80 Events	Age ≥80 Total	Age <80 Events	Age <80 Total	Weight	Odds Ratio M-H, Random, 95% CI
**1.5.1 2014-2018**						
Alawieh 2018	22	108	100	227	5.0%	0.32 [0.19, 0.56]
Azkune 2016	16	31	32	50	2.5%	0.60 [0.24, 1.49]
Broussalis 2015	5	21	66	138	2.0%	0.34 [0.12, 0.98]
Castonguay 2015	18	66	113	249	4.5%	0.45 [0.25, 0.82]
Cohen 2016	3	14	27	47	1.2%	0.20 [0.05, 0.82]
Figueiredo 2017	21	35	69	106	3.1%	0.80 [0.37, 1.76]
Imahori 2017	15	36	25	44	2.6%	0.54 [0.22, 1.32]
Karhi 2018	10	37	84	162	3.1%	0.34 [0.16, 0.76]
Kleine 2015	5	40	47	85	2.0%	0.12 [0.04, 0.32]
Koizumi 2018	27	78	73	143	4.7%	0.51 [0.29, 0.90]
Parrilla 2014	4	29	58	102	1.8%	0.12 [0.04, 0.37]
Sallustio 2017	19	62	54	157	4.2%	0.84 [0.45, 1.59]
Son 2017	15	34	108	173	3.4%	0.48 [0.23, 1.00]
Tajima 2017	11	25	34	53	2.3%	0.44 [0.17, 1.16]
**Subtotal (95% CI)**		**616**		**1736**	**42.3%**	**0.42 [0.32, 0.55]**
Total events	191		890			
Heterogeneity: Tau² = 0.11; Chi² = 21.87, df = 13 (P = 0.06); I² = 41%						
Test for overall effect: Z = 6.19 (P < 0.00001)						
**1.5.2 2019-2023**						
Adrien 2020	71	350	477	1046	8.3%	0.30 [0.23, 0.40]
Alawieh 2019	65	310	398	905	8.0%	0.34 [0.25, 0.46]
Choi 2021	8	16	25	44	1.7%	0.76 [0.24, 2.39]
Han 2023	48	148	712	1543	7.2%	0.56 [0.39, 0.80]
Jiao 2022	11	42	51	106	3.1%	0.38 [0.17, 0.84]
Kawabata 2019	6	19	16	40	1.7%	0.69 [0.22, 2.20]
Narloch 2023	5	34	37	128	2.1%	0.42 [0.15, 1.18]
Rezai 2019	16	57	63	136	3.9%	0.45 [0.23, 0.88]
Rhiner 2023	16	75	90	172	4.2%	0.25 [0.13, 0.46]
Scopelliti 2023	21	101	127	284	5.1%	0.32 [0.19, 0.55]
Sharobeam 2019	20	71	61	110	4.1%	0.32 [0.17, 0.60]
Sudre 2021	100	357	388	643	8.4%	0.26 [0.19, 0.34]
**Subtotal (95% CI)**		**1580**		**5157**	**57.7%**	**0.35 [0.29, 0.42]**
Total events	387		2445			
Heterogeneity: Tau² = 0.03; Chi² = 17.42, df = 11 (P = 0.10); I² = 37%						
Test for overall effect: Z = 11.08 (P < 0.00001)						
**Total (95% CI)**		**2196**		**6893**	**100.0%**	**0.38 [0.33, 0.45]**
Total events	578		3335			
Heterogeneity: Tau² = 0.06; Chi² = 42.95, df = 25 (P = 0.01); I² = 42%						
Test for overall effect: Z = 11.52 (P < 0.00001)						
Test for subgroup differences: Chi² = 0.97, df = 1 (P = 0.33), I² = 0%						

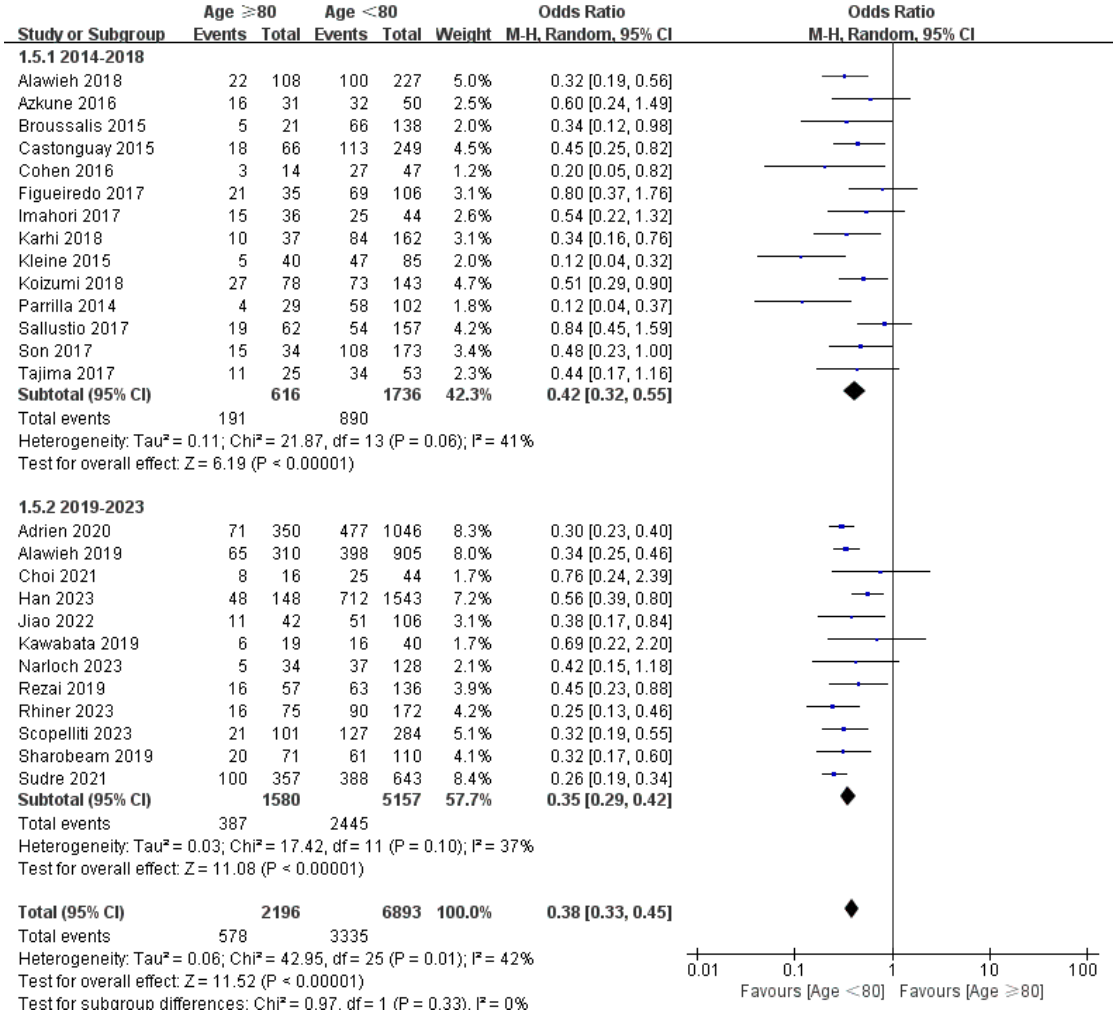

Supplemental Figure S3: Forest plot of subgroup analyses of mortality according to study design

Study or Subgroup	Age ≥80 Events	Age ≥80 Total	Age <80 Events	Age <80 Total	Weight	Odds Ratio M-H, Random, 95% CI
**1.7.1 single-center**						
Azkune 2016	6	31	5	50	2.4%	2.16 [0.60, 7.80]
Broussalis 2015	4	21	3	138	1.8%	10.59 [2.18, 51.39]
Choi 2021	0	16	4	44	0.6%	0.27 [0.01, 5.35]
Cohen 2016	6	15	4	55	2.0%	8.50 [1.99, 36.24]
Figueiredo 2017	5	35	9	106	2.8%	1.80 [0.56, 5.77]
Imahori 2017	3	36	2	44	1.4%	1.91 [0.30, 12.10]
Jiao 2022	15	42	15	106	4.1%	3.37 [1.46, 7.77]
Karhi 2018	17	37	16	162	4.1%	7.76 [3.39, 17.74]
Kleine 2015	13	40	10	85	3.6%	3.61 [1.42, 9.19]
Narloch 2023	9	34	10	128	3.4%	4.25 [1.56, 11.53]
Parrilla 2014	12	34	20	116	4.0%	2.62 [1.12, 6.14]
Rezai 2019	19	57	19	136	4.7%	3.08 [1.48, 6.41]
Rhiner 2023	43	75	51	172	5.7%	3.19 [1.82, 5.60]
Sallustio 2017	25	62	46	157	5.4%	1.63 [0.88, 3.01]
Sharobeam 2019	19	71	18	110	4.7%	1.87 [0.90, 3.87]
Son 2017	1	34	20	173	1.1%	0.23 [0.03, 1.79]
Sudre 2021	132	357	73	643	7.3%	4.58 [3.31, 6.34]
Tajima 2017	2	25	6	53	1.6%	0.68 [0.13, 3.64]
**Subtotal (95% CI)**		**1022**		**2478**	**60.6%**	**2.92 [2.14, 3.98]**
Total events	331		331			
Heterogeneity: Tau² = 0.19; Chi² = 34.50, df = 17 (P = 0.007); I² = 51%						
Test for overall effect: Z = 6.78 (P < 0.00001)						
**1.7.2 multicenter**						
Adrien 2020	178	350	229	1050	7.7%	3.71 [2.87, 4.79]
Alawieh 2018	37	108	45	227	6.0%	2.11 [1.26, 3.52]
Alawieh 2019	118	310	184	920	7.6%	2.46 [1.86, 3.25]
Castonguay 2015	29	66	68	249	5.7%	2.09 [1.19, 3.65]
Han 2023	28	148	243	1543	6.6%	1.25 [0.81, 1.93]
Kawabata 2019	5	19	11	40	2.5%	0.94 [0.27, 3.24]
Koizumi 2018	6	78	11	143	3.2%	1.00 [0.36, 2.82]
**Subtotal (95% CI)**		**1079**		**4172**	**39.4%**	**1.99 [1.38, 2.87]**
Total events	401		791			
Heterogeneity: Tau² = 0.16; Chi² = 25.24, df = 6 (P = 0.0003); I² = 76%						
Test for overall effect: Z = 3.67 (P = 0.0002)						
**Total (95% CI)**		**2101**		**6650**	**100.0%**	**2.51 [1.98, 3.18]**
Total events	732		1122			
Heterogeneity: Tau² = 0.17; Chi² = 64.88, df = 24 (P < 0.0001); I² = 63%						
Test for overall effect: Z = 7.66 (P < 0.00001)						
Test for subgroup differences: Chi² = 2.45, df = 1 (P = 0.12), I² = 59.1%						

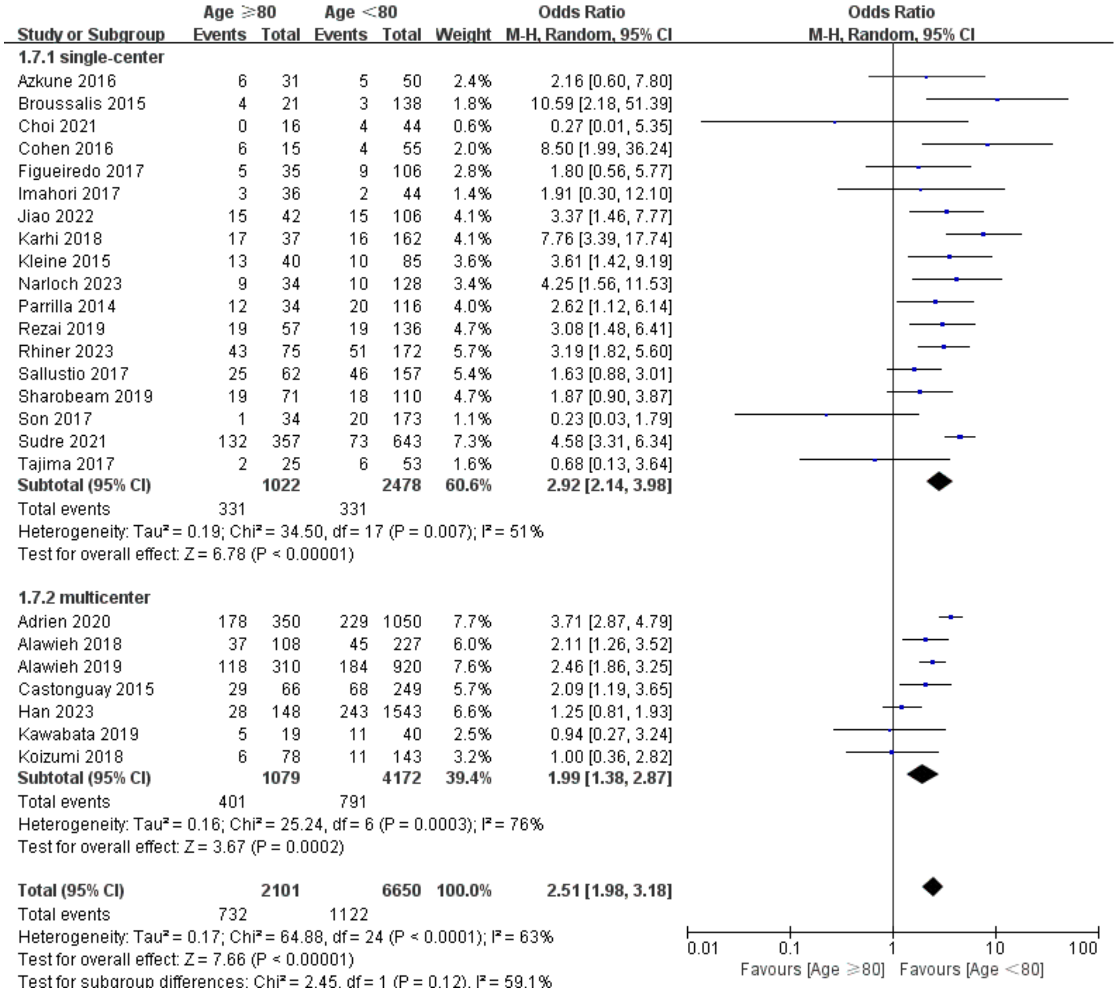

Supplemental Figure S4: Forest plot of subgroup analyses of functional outcome according to study period

Study or Subgroup	Age ≥80 Events	Age ≥80 Total	Age <80 Events	Age <80 Total	Weight	Odds Ratio M-H, Random, 95% CI
**1.8.1 2014-2018**						
Alawieh 2018	37	108	45	227	6.0%	2.11 [1.26, 3.52]
Azkune 2016	6	31	5	50	2.4%	2.16 [0.60, 7.80]
Broussalis 2015	4	21	3	138	1.8%	10.59 [2.18, 51.39]
Castonguay 2015	29	66	68	249	5.7%	2.09 [1.19, 3.65]
Cohen 2016	6	15	4	55	2.0%	8.50 [1.99, 36.24]
Figueiredo 2017	5	35	9	106	2.8%	1.80 [0.56, 5.77]
Imahori 2017	3	36	2	44	1.4%	1.91 [0.30, 12.10]
Karhi 2018	17	37	16	162	4.1%	7.76 [3.39, 17.74]
Kleine 2015	13	40	10	85	3.6%	3.61 [1.42, 9.19]
Koizumi 2018	6	78	11	143	3.2%	1.00 [0.36, 2.82]
Parrilla 2014	12	34	20	116	4.0%	2.62 [1.12, 6.14]
Sallustio 2017	25	62	46	157	5.4%	1.63 [0.88, 3.01]
Son 2017	1	34	20	173	1.1%	0.23 [0.03, 1.79]
Tajima 2017	2	25	6	53	1.6%	0.68 [0.13, 3.64]
**Subtotal (95% CI)**		**622**		**1758**	**45.2%**	**2.36 [1.61, 3.46]**
Total events	166		265			
Heterogeneity: Tau² = 0.24; Chi² = 27.29, df = 13 (P = 0.01); I² = 52%						
Test for overall effect: Z = 4.40 (P < 0.0001)						
**1.8.2 2019-2023**						
Adrien 2020	178	350	229	1050	7.7%	3.71 [2.87, 4.79]
Alawieh 2019	118	310	184	920	7.6%	2.46 [1.86, 3.25]
Choi 2021	0	16	4	44	0.6%	0.27 [0.01, 5.35]
Han 2023	28	148	243	1543	6.6%	1.25 [0.81, 1.93]
Jiao 2022	15	42	15	106	4.1%	3.37 [1.46, 7.77]
Kawabata 2019	5	19	11	40	2.5%	0.94 [0.27, 3.24]
Narloch 2023	9	34	10	128	3.4%	4.25 [1.56, 11.53]
Rezai 2019	19	57	19	136	4.7%	3.08 [1.48, 6.41]
Rhiner 2023	43	75	51	172	5.7%	3.19 [1.82, 5.60]
Sharobeam 2019	19	71	18	110	4.7%	1.87 [0.90, 3.87]
Sudre 2021	132	357	73	643	7.3%	4.58 [3.31, 6.34]
**Subtotal (95% CI)**		**1479**		**4892**	**54.8%**	**2.65 [1.95, 3.60]**
Total events	566		857			
Heterogeneity: Tau² = 0.15; Chi² = 34.84, df = 10 (P = 0.0001); I² = 71%						
Test for overall effect: Z = 6.26 (P < 0.00001)						
**Total (95% CI)**		**2101**		**6650**	**100.0%**	**2.51 [1.98, 3.18]**
Total events	732		1122			
Heterogeneity: Tau² = 0.17; Chi² = 64.88, df = 24 (P < 0.0001); I² = 63%						
Test for overall effect: Z = 7.66 (P < 0.00001)						
Test for subgroup differences: Chi² = 0.22, df = 1 (P = 0.64), I² = 0%						

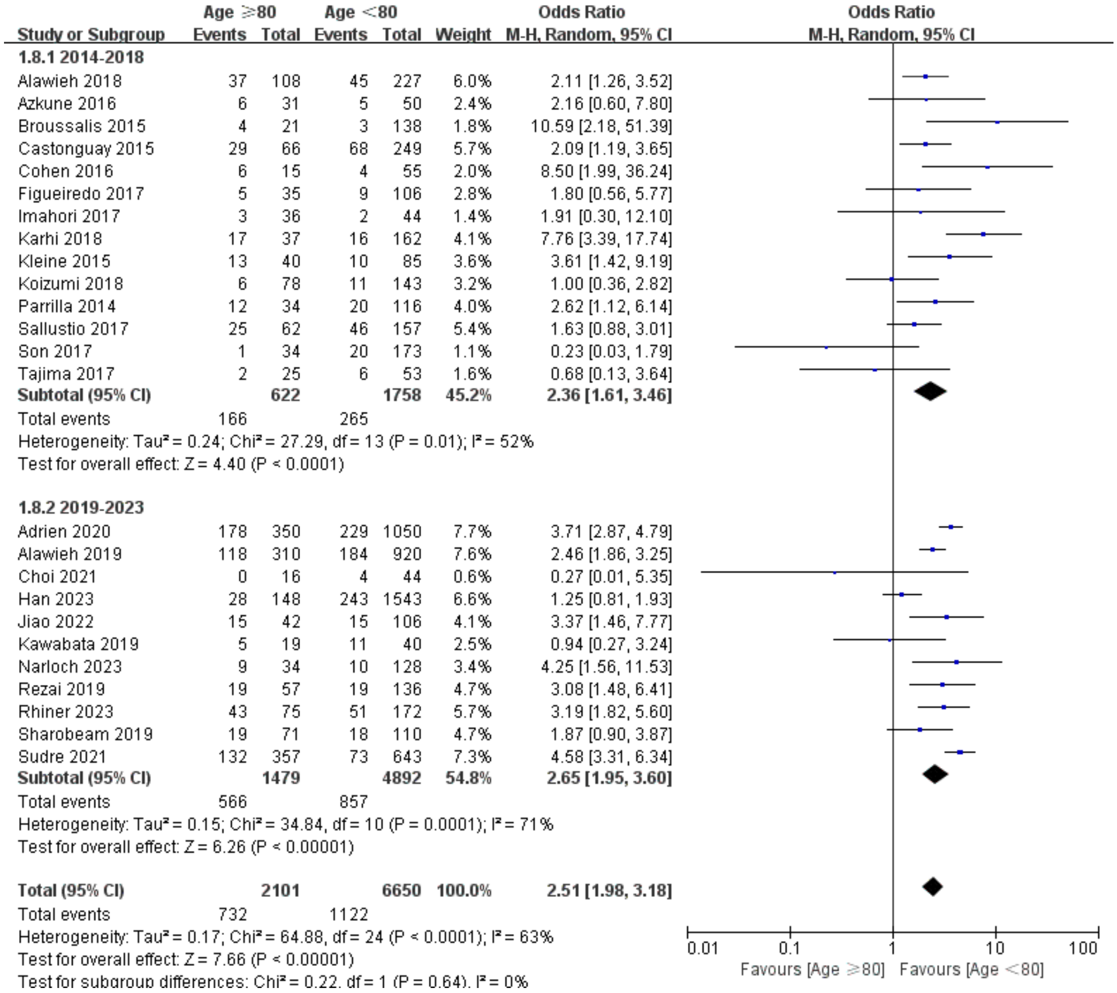

Supplemental Figure S5: Funnel plots of included studies for functional outcome.

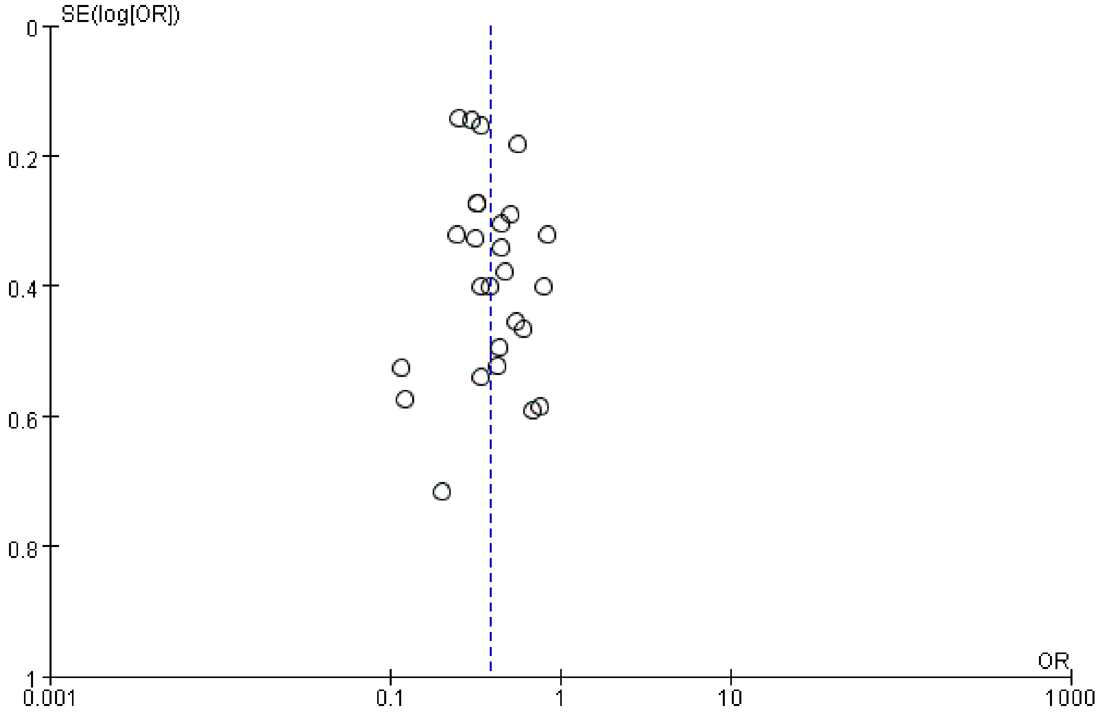

Supplemental Figure S6: Funnel plots of included studies for successful recanalization rate.

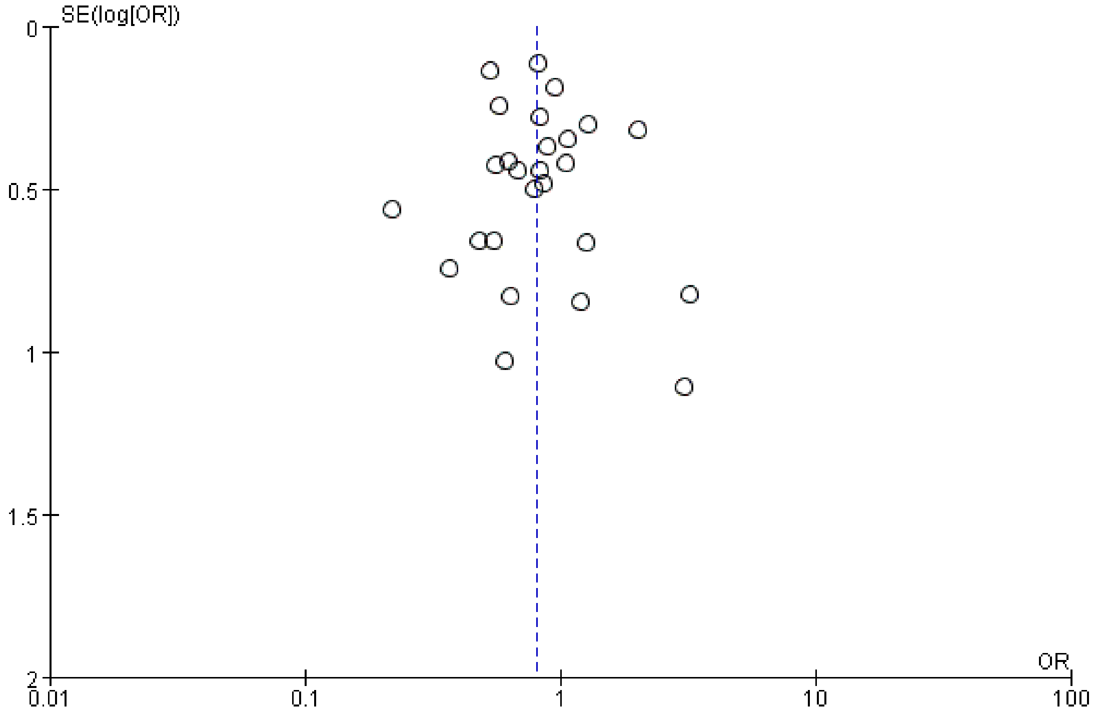

Supplemental Figure S7: Funnel plots of included studies for sICH.

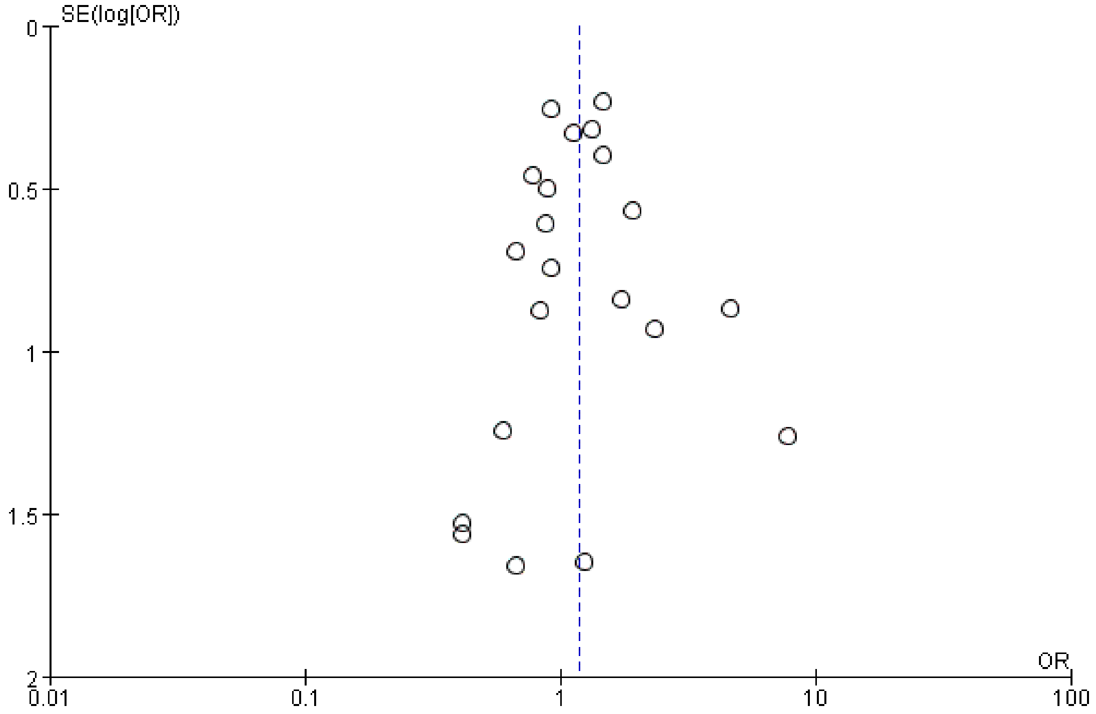

Supplemental Figure S8: Funnel plots of included studies for mortality.

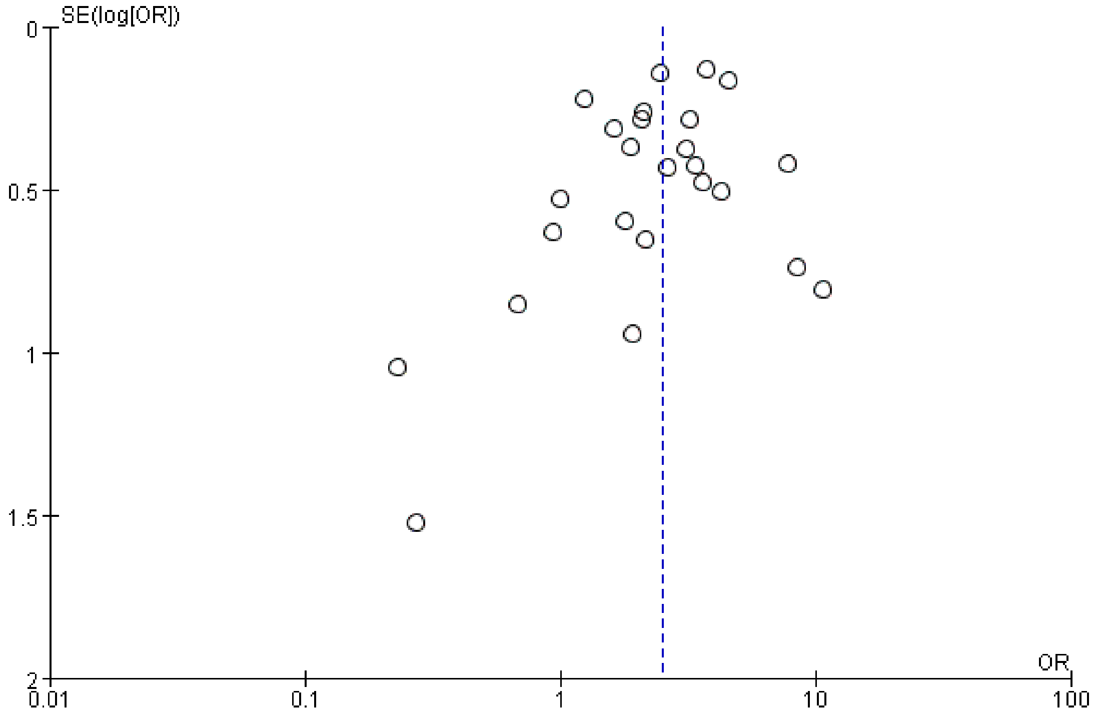

Supplement: Supplementary file 1 [file Data_Sheet_1.PDF]
